# Higher incidence of catheter-related bloodstream infection in femoral venous access than in subclavian venous access in the presence of tracheostomy

**DOI:** 10.1186/cc9648

**Published:** 2011-03-11

**Authors:** L Lorente, S Palmero, JJ Jiménez, I Roca, C Naranjo, J Castedo, S Huidobro, L Lorenzo, JL Iribarren, ML Mora

**Affiliations:** 1 Hospital Universitario de Canarias, La Laguna, Spain

## Introduction

A higher incidence of catheter-related bloodstream infection (CRBSI) in femoral than in subclavian catheter sites has been found [1,2]. Different guidelines for the prevention of CRBSI recommend avoiding femoral venous access sites [3,4]. However, the incidence of CRBSI in subclavian sites in the presence of tracheostomy is higher than without tracheostomy [5,6]. In addition, the incidence of CRBSI in jugular sites with tracheostomy is higher than in femoral sites [7]. Currently, there are no comparative data on the incidence of CRBSI between the femoral venous and the subclavian venous catheter site in the presence of tracheostomy and there are no recommendations in the guidelines relating to this circumstance; and this was the objective of the present study.

## Methods

A prospective observational 6-year study was carried out in the ICU of the University Hospital of the Canary Islands (Tenerife, Spain). We included all patients undergoing insertion of subclavian venous catheter in the presence of tracheostomy (subclavian-CVC+tracheo) or femoral venous catheter (femoral-CVC).

## Results

We diagnosed 26 CRBSI in 313 femoral-CVC during 2,565 days (10.1 CRBSI episodes/1,000 catheter-days) and five CRBSI in 147 subclavian-CVC+tracheo during 1,268 days (3.9 CRBSI episodes/1,000 catheter-days). Subclavian-CVC+tracheo showed a lower incidence of CRBSI than femoral-CVC (OR = 0.39; 95% CI = 0.001 to 0.91; *P *= 0.03). Survival analysis showed that subclavian-CVC+tracheo had greater CRBSI-free time than femoral-CVC (chi-square = 4.69; *P *= 0.03).

## Conclusions

Subclavian-CVC+tracheo could be considered a safer venous access site than femoral-CVC to minimize the risk of CRBSI.
